# Empirical analysis of socio-economic determinants of maternal health services utilisation in Burundi

**DOI:** 10.1186/s12884-021-04162-0

**Published:** 2021-10-07

**Authors:** Desire Habonimana, Neha Batura

**Affiliations:** 1 Faculty of Medicine, University of Burundi, Bujumbura, Burundi; 2 Institute for Global Health, University College London, London, UK

**Keywords:** Maternal health, Neonatal health, Antenatal care, Socio-economic determinants, Burundi

## Abstract

**Background:**

Timely and appropriate health care during pregnancy and childbirth are the pillars of better maternal health outcomes. However, factors such as poverty and low education levels, long distances to a health facility, and high costs of health services may present barriers to timely access and utilisation of maternal health services. Despite antenatal care (ANC), delivery and postnatal care being free at the point of use in Burundi, utilisation of these services remains low: between 2011 and 2017, only 49% of pregnant women attended at least four ANC visits. This study explores the socio-economic determinants that affect utilisation of maternal health services in Burundi.

**Methods:**

We use data from the 2016–2017 Burundi Demographic and Health Survey (DHS) collected from 8941 women who reported a live birth in the five years that preceded the survey. We use multivariate regression analysis to explore which individual-, household-, and community-level factors determine the likelihood that women will seek ANC services from a trained health professional, the number of ANC visits they make, and the choice of assisted childbirth.

**Results:**

Occupation, marital status, and wealth increase the likelihood that women will seek ANC services from a trained health professional. The likelihood that a woman consults a trained health professional for ANC services is 18 times and 16 times more for married women and women living in partnership, respectively. More educated women and those who currently live a union or partnership attend more ANC visits than non-educated women and women not in union. At higher birth orders, women tend to not attend ANC visits. The more ANC visits attended, and the wealthier women are; the more likely they are to have assisted childbirth. Women who complete four or more ANC visits are 14 times more likely to have an assisted childbirth.

**Conclusions:**

In Burundi, utilisation of maternal health services is low and is mainly driven by legal union and wealth status. To improve equitable access to maternal health services for vulnerable population groups such as those with lower wealth status and unmarried women, the government should consider certain demand stimulating policy packages targeted at these groups.

## Background

In low- and middle-income countries (LMICs), women often have poor health outcomes during pregnancy, childbirth, and the postpartum period [1]. These outcomes include severe bleeding, infections, high blood pressure, delivery complications, unsafe abortion, and the aggravation of pre-existing health conditions [2, 3]. A recent systematic review found that of all causes of maternal deaths, haemorrhage accounts for 27.1% of deaths while hypertension and sepsis are responsible for 14 and 10.7% of deaths, respectively [3]. Of about 830 women who die from pregnancy- or childbirth-related complications around the world every day, 99% occur in LMICs [2]. It is also estimated that of the 2.6 million stillbirths occurring globally in 2015, 98% were in LMICs [4]. Further, the risk of a woman in a LMIC dying from a maternal-related cause during her lifetime is about 33 times higher compared to her counterpart in a high-income country [5]. Specifically, such a risk remains high in resource-constrained settings such as Burundi [6]. It is estimated that about two Burundian women out of 100 will die of pregnancy-related conditions including haemorrhage, infections, eclampsia and unsafe abortion during their reproductive life [7]. Despite having made progress in maternal health indicators during the past three decades, Burundi has one of the highest maternal mortality ratios in the world: 712 maternal deaths per 100,000 live births which is the eighth highest in the world [2].

Most health conditions occurring during pregnancy, childbirth, and in the post-partum period are preventable and curable by timely and appropriate maternal health care [4, 8]. Evidence has shown that provision of quality antenatal, delivery and postnatal care is an effective strategy to avert maternal and neonatal deaths [4, 9, 10]. With an aim to improve antenatal care (ANC), the World Health Organisation (WHO) introduced the Focused ANC (FANC) model which recommends a minimum of four ANC visits during pregnancy. According to the FANC guidelines, the first and second ANC visits should take place during the first and second trimesters, respectively; and the last two visits during the third trimester of pregnancy [11]. The FANC model was updated in 2016 and recommends a minimum of eight ANC contacts between the pregnant woman and a skilled health provider during pregnancy. Instead of “visit”, the 2016 model uses the word “contact” to imply that a pregnant woman makes an active connection with a health care provider, which is more likely to improve the woman’s experience of care. The first contact is scheduled to happen during the first trimester, the second and third contacts during the second trimester, and the remaining five contacts during the third trimester of the pregnancy [12, 13]. Both the models emphasise that births are assisted by skilled birth attendants, preferably at a health facility [13].

Despite these recommendations, maternal health indicators remain low across the East African subregion (Table 1). In Burundi, a low-income and politically fragile country located astride East and Central Africa, pregnant women have received full free maternal health services since 2006 [19], and frontline health care providers receive performance-based incentives to deliver care [20]. However, these policy solutions have not improved the utilisation of maternal health services: the proportion of pregnant women who attend at least four ANC visits (and most importantly eight contacts) and those delivering in a health facility, which remain well below national targets [14].

**Table 1:** Sub-regional performance in maternal health indicators in East Africa

	Women attending at least 4 ANC visits	Births assisted by skilled birth attendant
Burundi	49%	85%
Kenya	58%	62%
Rwanda	44%	91%
Tanzania	51%	63%
Uganda	60%	74%

Source: National DHS surveys [14–18]

Empirical evidence from LMICs indicates that women’s utilisation of health services is affected by several social and economic factors at the individual-, household- and community-level [21, 22]. In a study of the socio-economic determinants of maternal health care utilisation among Turkish women, findings suggested that more educated women and those with lower parity were more likely to seek ANC services from a qualified health professional and to deliver in a health facility [21]. In the same way, a recent meta-analysis study by Finlayson and Downe (2013) found that the lack of decision-making power and the absence of perceived ANC attendance benefits are the key barriers to health seeking among pregnant women [23]. Additionally, in their study of the socio-economic determinants of maternal health care utilisation in Ghana, Patience Aseweh Abor et al. (2011) found significant impact of the mother’s age, education level, economic status, ethnicity, religious affiliation, residence and location on her maternal health care seeking behavior [24]. According to the same study, the type of pregnancy, single or twin, also affects the mother’s decision both during pregnancy and childbirth [24]. Other studies have found similar results in different contexts including in Ghana [25], Nepal [26], Benin, Burkina Faso and Cameroon [27].

At the household level, a wealth of literature finds that women from large and poor families underutilise maternal health services. This was found by studies in the Philippines [28], India [29], Bangladesh [30], Ghana [24], Turkey [21], as well as in Nepal and Nigeria [31, 32]. Family size has also been found to be associated with maternal health services’ utilisation [33]. At the community level, being from a rural setting is negatively associated with maternal health services’ utilisation. In Thailand for example, the probability that a woman delivers in a health facility and receives childbirth assistance by a skilled birth attendant was significantly reduced by long travel distances to the health facility and the mountainous terrain while maternal health services utilisation was higher among women living closer to a health facility [34]. Similarly, in a study by Navaneetham and Dharmalingam (2002), the place of residence determined utilisation of maternal health services among women in the states of Andhra Pradesh, Karnataka, Kerala and Tamil Nadu of south India. According to findings from this study, the likelihood of institutional delivery increased by over two times among women in urban settings of Andhra Pradesh, Tamil Nadu and Karnataka and there was a significant difference in the place of delivery between women in rural versus urban areas of Kerala [35].

Evidence from the East Africa sub-region, specifically from Kenya, Rwanda, Tanzania, and Uganda presents similar findings. More educated women, those from wealthier families, and those living in cities were found to be more likely to utilise maternal health services in Uganda [36] and in Kenya [37]. Moreover, access to health insurance, exposure to media and higher community-level socioeconomic status were associated with a higher likelihood of seeking maternal health services among Kenyan women [37]. In a study to understand Rwandan mothers’ perceptions and experiences of using maternal health care, poverty, sociocultural believes, and long distances to a health facility emerged as key factors leading to suboptimal utilisation of maternal health care services [38]. Similarly, long distances to a clinic affect women’s maternal health care seeking behavior in Tanzania [39].

However, there is a scarcity of published evidence from the other East African countries such as Burundi and South Sudan, where maternal service utilisation remains persistently low. Specifically in the context of Burundi, to our knowledge, there is no study that explores why pregnant women underutilise maternal health services, which are already government-subsidised and provided free of charge. Therefore, this study aims to fill this gap by investigating the socio-economic factors affecting the likelihood of women attending ANC services provided by a trained health professional, the number of ANC visits attended, and the choice of type of delivery in Burundi.

## Methods

### Data and variables

We use data from the nationally representative 2016–2017 Burundi Demographic and Health Survey (DHS). The study sample consists of 8941 Burundian women who reported at least one live birth in the five years preceding the survey. For women who had multiple births, we consider the data for the most recent pregnancy with the view of minimising recall bias.

The dependent variables used for analyses are presented in Table 2. These are utilisation of ANC services provided by a qualified health provider, the number of ANC visits, and the place of delivery and birth assistance. These variables are key indicators for the monitoring of maternal health care [40–42].

**Table 2:** Dependent variables

Dependent variable	Variable management
**Outcome 1**. Utilisation of ANC services provided by a qualified health provider	Dummy variable **= 1** if ANC services provided by a medical doctor, a nurse or a midwife were used; and = **0** otherwise
**Outcome 2**. Number of ANC visits	Continuous variable capturing the number of ANC visits outcome attended during pregnancy
**Outcome 3**. Assisted delivery	Dummy variable = **0** for an unassisted home delivery; = **1** for a home delivery assisted by a skilled birth attendant; = **2** for delivery in a health facility

We included individual-, household-, and community-level independent variables identified by a review of literature and an understanding of the country context (Table 3).

**Table 3:** Independent variables

Explanatory variables	Sample (***N*** = 8941)	
	n	%
**Individual-level characteristics**		
*Age*		
15–19 years	232	2.60
20–34 years	6131	68.57
35–49 years	2578	28.83
*Birth order*		
1st child	1498	16.75
2nd-3rd child	3034	33.94
4th–5th child	2218	24.81
6th child and above	2191	24.50
*Education*		
None	4118	46.06
Primary	3790	42.39
Secondary	964	10.78
Tertiary	69	0.77
*Occupation*		
No	668	07.47
Yes	8273	92.53
*Religion*		
Catholic	5062	56.62
Protestant	3352	37.49
Muslim	294	3.29
Traditional	233	2.61
*Health insurance*		
No	6778	75.81
Yes	2163	24.19
*Marital status*		
Never married	364	4.07
Legal union	5796	64.82
Partnership	2056	22.99
Divorced/separated/widowed	725	8.13
*Decision-making power*		
No	7514	84.04
Yes	1427	15.96
**Household-level characteristics**		
*Family size*		
1–5 individual(s)	4396	49.17
> 5 individuals	4545	50.83
*Husband’s education level*		
None	4134	46.24
Primary	3875	43.34
Secondary	792	8.86
Tertiary	140	1.56
*Husband’s occupation*		
No	1646	18.41
Yes	7295	81.59
*Child’s sex*		
Female	4378	48.96
Male	4563	51.04
*Income quintile*		
First (poorest)	2043	22.85
Second	1913	21.40
Thirds	1791	20.04
Fourth	1653	18.49
Fifth (richest)	1540	17.23
**Community-level characteristics**		
*Residence*		
Urban	980	9.84
Rural	8061	90.16
*Easy access to a health facility*		
No	2297	32.73
Yes	6014	67.27

In most cases, we did not need to recode or recategorize the independent variables. However, we did need to recategorize some variables for ease of analysis and precision of estimates. The age of women was categorised as follows: between 15 and 19 years (adolescents), between 20 and 34 years (youth) and between 35 and 49 years (adults). This definition is consistent the African Youth Charter (AYC) which is a legal continental framework used in most of African countries [43]. The birth order was categorised as follows: first, second to third, fourth to fifth, and more than sixth birth orders. The original dataset categorised religion into eight categories. These were collapsed into four namely: Catholic, Protestant, Muslim, and traditional practitioners, to account for the demographic composition of the population and to avoid an enlargement of the error term in the regression due to large differences in values between categories. Marital status was also coded into four categories: women who never lived in union, those who currently live in legal union, those currently living in partnership, and women who ever lived in union but are currently living alone. In the fourth category of women, we combined widows, divorced women, and those who were separated because they have one civil status in common - the transition out of union.

For the family size variable, we based our categories on Burundi’s family and reproductive health advice. According to this, couples should have a maximum of three children. Therefore, the family size took a value of 1 for families whose household did not exceed five individuals (three children and two parents), and 2 for households of more than five individuals. All the 18 provinces of Burundi were included in the analysis.

### Model specification

#### ANC utilisation

We use logistic regression to estimate the following empirical model to understand women’s likelihood of utilising ANC services provided by a trained health professional, controlling for individual-, household-, and community-level characteristics:

1 $$\log \left[\frac{p_{i1}}{p_{i0}}\right]={\beta}_0+{\beta}_1{X}_{ij}+{\beta}_2{Y}_{ij}+{\beta}_3{Z}_j+{\varepsilon}_{ij}^1$$

Here, the dependent variable is the log odds that a woman ***i*** will choose alterative ***j*** relative to alternative **0**, where **0** = non-use of ANC services from a trained provider; and **1** = consultation with a medical doctor, nurse or midwife. Independent variables are grouped into three categories; namely individual-level factors represented by a standard vector of covariates ***X***, household-level determinants corresponding to the standard vector of covariates ***Y***, and community-level determinants represented by the standard vector of covariates ***Z***. The model includes a dummy variable that captures provincial effects. ***β***_**0**_ captures fixed effects and ***β***_**1,2*,*3**_ detect random effects on the probabilities of using ANC services from a trained provider.

### Number of ANC visits

We then estimate the effect of individual-, household-, and community-level factors on the number of ANC visits using linear regression. The empirical model is specified as:

2 $$anc\_{visits}_i={\beta}_0+{\beta}_1{X}_{ij}+{\beta}_2{Y}_{ij}+{\beta}_3{Z}_j+{\varepsilon}_{ij}^1$$

Where, the outcome *anc* _ *visits*_*i*_ is continuous and represents the number of ANC visits that a woman ***i*** attends during her pregnancy. ***X,Z,*** and ***Y*** are the same standard vectors used in the logistic model. This model concerns women who reported attending one or more ANC visits. For the linear regression model, we used 95% *p*-value to ascertain significance of coefficients.

### Childbirth service utilisation

We use a multinomial logistic model to explore women’s likelihood of seeking delivery services from a trained birth attendant. The empirical model is given by:

3 $$\log \left[\frac{p_{ij}}{p_{i0}}\right]={\beta}_0+{\beta}_{1j}{X}_{ij}+{\beta}_{2j}{Y}_{ij}+{\beta}_{3j}{Z}_j+{\varepsilon}_{ij}^2$$

Where the response variable is the log odds that a woman ***i*** will choose delivery alterative ***j*** (***j =*** **1**, **2**) relative to **0**, where **0** = home delivery without assistance by a skilled birth attendant; **1** = home delivery with assistance by a skilled birth attendant; and **2** = health facility delivery. Independent variables are represented by standard vectors of covariates ***X,Y,*** and ***Z*** used in previous models. In addition to standard covariates, this model includes the number of ANC visits as this has been found to positively predict assisted delivery [44].

All models assume that community-level effects are invariant for women living in the same setting. With an aim to attempt validate the assumption, we use two variables to account for province and residence. Residence is binary; rural versus urban; and there are 18 provinces each having a rural and an urban component. As such, community-level factors are assumed to be constant for women living in residence ***i*** within province ***j***.

All discrete models used 95% confidence intervals to ascertain significance of coefficients, which the literature claims to be more reliable [30, 45].

### Patient and public involvement

This study used DHS datasets. No patients or members of the public were involved in the design, analysis or reporting of this study.

## Results

### Utilisation of ANC services provided by a trained health professional

A woman’s occupation and household wealth have significant positive impact on the likelihood of utilising ANC services from a trained health professional (Table 4). Women who are employed, and those belonging to the third income quintile are more than three times more likely (OR = 3.407) to utilise ANC services from a trained provider. The likelihood that a woman consults a trained health provider for ANC during pregnancy is 18 times (OR = 18.334) higher for married women, 16 times (OR = 15.857) for women in partnership and four times (OR = 4.410) for single women who ever lived in partnership or in union compared never married women. Women living in Kirundo or Mwaro province are two times less likely to utilise ANC services from a trained health professional.

**Table 4:** Regression results: likelihood of using ANC services

Explanatory variables			Sample (***N*** = 8941)	
	Coefficient	OR	95% CI	p-value
*Age (reference:* 15–19 *years)*				
20–34 years	−0.479	0.620	0.134–2.870	0.540
35–49 years	−1.650	0.192	0.350–1.057	0.058
*Birth order (reference: 1st child)*				
2nd-3rd child	−0.203	0.817	0.350–1.907	0.639
4th–5th child	0.098	1.103	0.396–3.076	0.850
6th child and above	0.202	1.223	0.456–3.281	0.688
*Education (reference: none)*				
Primary	0.442	1.557	0.869–2.788	0.137
Secondary	1.527	4.604	0.820–25.853	0.083
Tertiary	–	–	–	
*Occupation (reference: no)*				
**Yes**	**1.226**	**3.407**	**1.553–7.473**	**0.002**
*Religion (reference: Catholic)*				
Protestant	−0.270	0.763	0.446–1.307	0.324
Muslim	−0.753	0.471	0.054–4.080	0.493
Traditional	−0.012	0.988	0.227–4.307	0.987
*Health insurance (reference: no)*				
Yes	0.472	1.604	0.716–3.590	0.250
*Marital status (reference: never married)*				
**Legal union**	**2.909**	**18.334**	**4.526–74.302**	**0.000**
**Partnership**	**2.764**	**15.857**	**3.531–71.219**	**0.000**
**Divorced/separated/widowed**	**1.484**	**4.410**	**1.634–11.904**	**0.003**
*Decision-making power (reference: no)*				
Yes	0.182	1.120	0.517–2.787	0.671
*Family size (reference:* 1–5 *people)*				0.564
> 5 individuals	−0.171	0.843	0.471–1.509	
*Husband’s education (reference: none)*				
Primary	−0.192	0.826	0.425–1.602	0.570
Secondary	–	–	–	
Tertiary	–	–	–	
*Husband’s occupation (reference: none)*				
Yes	0.017	1.017	0.333–3.103	0.976
*Child’s sex (reference: Male)*				
Female	−0.451	0.637	0.386–1.053	0.079
*Income quintile (reference: first)*				
Second	0.504	1.655	0.855–3.200	0.134
**Third**	**1.106**	**3.021**	**1.287–7.092**	**0.011**
Fourth	0.753	2.124	0.903–4.992	0.084
Fifth	0.278	1.321	0.488–3.576	0.583
*Residence (reference: urban)*				
Rural	−0.873	0.419	0.107–1.630	0.208
*Easy access to a health facility (reference: no)*				
Yes	0.538	1.713	0.987–2.972	0.056
*Province*				
**Kirundo**	**−1.928**	**0.145**	**0.023–0.929**	**0.042**
**Mwaro**	**−2.003**	**0.135**	**0.019–0.941**	**0.043**

### Number of ANC visits

The number of ANC visits made by women is likely to increase with higher education status and for women who are currently in union or have previously been in union (Table 5). Compared with never married women, being in a current union or partnership corresponds to an increase of about 0.7 (*p* = 0.000) on average in the number of ANC visits; a previous union is associated with an increase of about 0.6 (*p* = 0.003) on average in the number of ANC visits. Women who achieved a tertiary education attend 1.3 (*p* = 0.000) more visits on average than women who did not have any schooling. Moreover, being a resident of Gitega, Kayanza or Kirundo province is associated with an increase of about 0.4 on average in the number of ANC visits. The number of ANC visits significantly decreases with higher birth orders. The number of ANC visits attended decreases by 0.2 (*p* = 0.006) on average for women at their second and third pregnancies, decreases by 0.4 (0.000) on average for the fourth and fifth pregnancies, and further halves 0.5 (*p* = 0.000) for women above the fifth pregnancy.

**Table 5:** Regression results: the number of ANC visits

Explanatory variables		Sample (***N*** = 8879)	
	Co-efficient	S.E.	95% p-value
*Age (reference:* 15–19 *years)*			
20–34 years	0.107	0.182	0.558
35–49 years	0.088	0.199	0.659
*Birth order (reference: 1st child)*			
**2nd-3rd child**	**−0.243**	**0.089**	**0.006**
**4th–5th child**	**−0.401**	**0.109**	**0.000**
**6th child and above**	**−0.520**	**0.130**	**0.000**
***Education (reference: none)***			
Primary	0.114	0.062	0.066
Secondary	0.0	0.116	0.722
**Tertiary**	**1.277**	**0.332**	**0.000**
*Occupation (reference: no)*			
Yes	−0.036	0.109	0.743
*Religion (reference: Catholic)*			
Protestant	0.051	0.061	0.403
Muslim	0.270	0.149	0.070
Traditional	0.291	0.187	0.119
*Health insurance (reference: no)*			
Yes	0.066	0.070	0.337
*Marital status (reference: never married)*			
**Legal union**	**0.714**	**0.196**	**0.000**
**Partnership**	**0.666**	**0.201**	**0.001**
**Divorced/separated/widowed**	**0.540**	**0.179**	**0.003**
*Decision-making power (reference: no)*			
Yes	0.119	0.076	0.116
*Family size (reference:* 1–5 *people)*			
> 5 individuals	0.092	0.074	0.213
*Husband’s education (reference: none)*			
Primary	0.012	0.067	0.854
Secondary	0.058	0.119	0.625
**Tertiary**	**0.446**	**0.236**	**0.059**
*Husband’s occupation (reference: none)*			
Yes	−0.072	0.111	0.520
*Child’s sex (reference: Male)*			
Female	0.017	0.541	0.747
*Income quintile (reference: first)*			
Second	−0.006	0.086	0.948
Third	0.137	0.090	0.127
Fourth	−0.006	0.095	0.950
Fifth	0.062	0.118	0.601
*Residence (reference: urban)*			
Rural	0.007	0.103	0.942
*Easy access to a health facility (reference: no)*			
Yes	−0.026	0.061	0.673
*Province*			
**Gitega**	**0.457**	**0.161**	**0.005**
**Kayanza**	**0.333**	**0.164**	**0.042**
**Kirundo**	**0.400**	**0.158**	**0.012**

### Place of delivery and birth assistance

None of the independent variables included in our analysis were significantly associated with the choice of a home delivery, assisted or not (Table 6). However, we find that the likelihood that a woman seeks birth assistance from a health facility is significantly determined by her parity, religion, wealth status, place of residence, access to a health facility and whether she attended ANC visits. The more ANC visits a woman attends, the more likely it is that she delivers in a health facility. Women who attend one to three ANC visits are eight times (OR = 8.341) more likely to seek birth assistance from a health facility compared to those women who did not attend any visits. Women who attend four or more ANC visits are 14 times (OR = 13.53) more likely to deliver in a health facility. The likelihood of assisted childbirth increases with wealth status and is nearly three times higher (OR = 2.617) for women who belong to the richest families. Having easy access to a health facility is associated with an increased likelihood of assisted childbirth (OR = 1.302). In contrast, rural women (OR = 0.352), those with higher parity (OR = 0.374 for women at their second and third pregnancies, OR = 0.313 for those at their fourth and fifth pregnancies, and OR = 0.300 for higher parities), those practicing traditional religion (OR = 0.559), and women from Bururi (OR = 0.230), Muramvya (OR = 0.490), and Rumonge (OR = 0.311) are less likely to deliver in a health facility.

**Table 6:** Regression results: the likelihood of choosing a delivery option

Base outcome: Home deliveries without assistance by a skilled birth attendant		
Explanatory variables	Sample (N = 8941)	
	Assisted home deliveries	Health facility deliveries
	OR [95% CI]	OR [95% CI]
*Age (reference:* 15–19 *years)*		
20–34 years	1.699 [0.534–5.408]	1.215 [0.576–2.560]
35–49 years	1.688 [0.496–5.746]	0.896 [0.401–2.002]
*Birth order (reference: 1st child)*		
2nd-3rd child	0.786 [0.454–1.363]	0.374 [0.244–0.572]
4th–5th child	0.613 [0.312–1.204]	0.313 [0.191–0.513]
6th child and above	0.646 [0.326–1.278]	0.300 [0.177–0.510]
*Education (reference: none)*		
Primary	0.953 [0.709–1.282]	1.189 [0.960–1.473]
Secondary	0.997 [0.463–2.143]	1.334 [0.741–2.404]
*Occupation (reference: no)*		
Yes	1.032 [0.528–2.017]	0.816 [0.494–1.347]
*Religion (reference: Catholic)*		
Protestant	0.853 [0.626–1.164]	0.969 [0.761–1.235]
Muslim	0.780 [0.230–2.031]	1.330 [0.591–2.996]
Traditional	0.899 [0.483–1.675]	0.559 [0.339–0.922]
*ANC visits (reference: 0 visit)*		
**1–3 visits**	1.982 [0.846–4.646]	**8.341 [3.936–17.673]**
**4 and more visits**	2.344 [0.988–5.560]	**13.53 [6.315–29.016]**
*Health insurance (reference: no)*		
Yes	0.836 [0.596–1.173]	1.114 [0.858–1.448]
*Marital status (reference: never married)*		
Legal union	2.008 [0.683–5.901]	1.823 [0.867–3.836]
Partnership	1.927 [0.647–5.740]	1.456 [0.676–3.130]
Divorced/separated/widowed	2.450 [0.974–6.163]	1.767 [0.890–3.507]
*Decision-making power (reference: no)*		
Yes	1.184 [0.826–1.697]	1.122 [0.860–1.333]
*Family size (reference:* 1–5 *people)*		
> 5 individuals	1.101 [0.761–1.593]	0.946 [0.724–1.235]
*Husband’s education (reference: none)*		
Primary	0.953 [0.714–1.272]	1.062 [0.847–1.333]
Secondary	0.921 [0.456–1.868]	1.441 [0.866–2.397]
*Husband’s occupation (reference: none)*		
Yes	0.877 [0.493–1.560]	1.116 [0.713–1.748]
*Child’s sex (reference: Male)*		
Female	0.939 [0.723–1.219]	0.896 [0.734–1.093]
*Income quintile (reference: first)*		
Second	0.760 [0.548–1.053]	1.133 [0.883–1.454]
**Third**	1.043 [0.714–1.523]	**1.453 [1.104–1.913]**
**Fourth**	1.087 [0.746–1.584]	**1.522 [1.124–2.063]**
**Fifth**	0.825 [0.397–1.716]	**2.617 [1.575–4.349]**
*Residence (reference: urban)*		
**Rural**	0.481 [0.762–1.347]	**0.352 [0.156–0.792]**
*Easy access to a health facility (reference: no)*		
**Yes**	1.013 [0.762–1.347]	**1.302 [1.055–1.608]**
*Province*		
Bururi	0.687 [0.253–1.868]	0.230 [0.143–0.627]
Muramvya	1.150 [0.468–2.824]	0.490 [0.241–0.999]
Rumonge	0.581 [0.222–1.519]	0.311 [0.134–0.722]

## Discussion

In this study, we use secondary data from the 2016–2017 DHS survey from Burundi to understand which socio-economic factors determine utilisation of maternal health services in the country. Our results indicate that women’s wealth, occupation, and marital status have a positive effect on the likelihood of utilising ANC services from a trained health professional. This is consistent with evidence from other LMICs. For instance, a study conducted in Madhya Pradesh, India found that wealthier women are more likely to seek ANC from a qualified professional [46]. Similar results were obtained in studies conducted in Nigeria [31] and Kenya [47]. In studies conducted in Nigeria, Cambodia, the Philippines and in Timor-Leste [31, 48], occupation was a significant predictor of the likelihood of seeking ANC from skilled providers. A Kenyan study found that married women were three times more likely to seek ANC services than their single counterparts [47]. The impact of marital status can be policy related. For instance, in Burundi, a predominantly Catholic country, access to ANC services in public health facilities is conditional on the provision of a legal marriage certificate [14]. This may prevent unmarried women from seeking ANC services.

We also find that a woman’s marital status and education have a positive impact on the number of ANC visits. Higher birth orders decrease the number of ANC visits. Additionally, we find that women from Gitega, Kayanza and Kirundo provinces have an advantage in terms of ANC visits. In support of our findings, high parity had also been found to predict poor maternal health care services’ utilisation in Bangladesh [30] and in Ethiopia [49]. A recent multi-country study conducted in Bangladesh, Burkina Faso, Ethiopia, Mali, Mozambique, Nepal and Niger found that mothers of higher parity were less likely to attend ANC visits [50]. Reasons might include the fact that women place high value to lower pregnancies and that health care workers strongly recommend institutional delivery for primiparous women [51]. Additionally, higher parity women may not feel the need for ANC visits drawing on previous experience of pregnancy and delivery [51, 52].

Our findings show that the number of ANC visits attended, higher wealth status, urbanicity and the accessibility to a health facility have a positive impact on the likelihood of seeking assisted health facility delivery. This is consistent with studies done in other LMIC settings. For example, a study set in Nigeria found that urban and wealthy women were about two times more likely to receive assisted delivery care [31]. In Ghana, compared with women from the least well-off families, the likelihood of delivering in a health facility increased from the poorer (OR = 0.159), the middle (OR = 0.325), the richer (OR = 0.807) to the richest (OR = 1.208) families [24]. In India, women who received ANC services were about four times more likely to deliver in a health facility and was two times more likely for urban than rural women [46]. In our study, the likelihood of assisted deliveries as a result of the number of ANC visits is higher than other findings observed in the literature. This can be attributed to the policy of Burundi whereby women who are eligible for ANC services are the ones eligible for delivery care. In our study, women who believe in traditional religion and those with higher parity are found to be less likely to deliver in a health facility. In fact, it has been established that traditional beliefs hinder maternal health care seeking behavior [53]. In a study that sought to understand the relevance of religion to maternal health services utilisation in Ghana; traditionalist women were twice less likely to deliver in a health facility and were 70% less likely to attend four ANC visits [53].

### Policy implications

Findings from our study feed into the local context. For example, in addition to the fact that husbands play an important traditional role in the family and Burundi’s society, today’s maternal health practice obliges a pregnant woman to be accompanied by her husband for each ANC visit [54]. Alternatively, a woman can be received for ANC services provided that she presents a marriage certificate alongside convincing evidence to justify the husband’s absence. Therefore, it turns out that married women and those living in partnership are likely to gain company from their husbands which further implies that unmarried women are less likely to attend ANC visits and deliver in a health facility. Most importantly, in this majority Christian country where illegal marriages are prohibited, delivery in a public health facility is determined by the provision of a marriage certificate. This also widens the gap between single and married women with regard to seeking of delivery services from a health facility. To provide equitable access of maternal health services to unmarried women, policy interventions could discontinue the requirement of a legal union certificate at the point of maternal health care. Other demand side stimulating mechanisms, such as cash transfers or vouchers that can address economic barriers to access and utilisation of services may also be implemented to increase utilisation amongst more vulnerable groups.

### Strengths and limitations

Our study has some strengths and limitations. The data used for analysis is sourced from a nationally representative survey collected using multistage sampling techniques. The dataset did not contain any missing values. Attempts to minimise recall bias were made by only including information about the most recent pregnancy. However, results of this study should be interpreted with some caution as the cross-sectional design does not allow us to understand causality but does offer evidence on associations that can guide policy. Using a longitudinal dataset that captures information on a cohort’s utilisation of health services and changes in their socio-economic circumstances would provide stronger evidence to guide policy. This could a be a direction for future research.

## Conclusions

While findings feed into general evidence, some are specifically meaningful for Burundi’s context. We found strong evidence of the effect of marital status of women on their utilisation of maternal health services. Maternal policy prevents unmarried women from seeking and accessing maternal health care services and delivery in a public or religious clinic is determined by a marriage certificate, which widens the gap between married and unmarried women. To improve ANC attendance and assisted health facility deliveries, our recommendation is a revisiting of the Burundi’s maternal health policy to enable single women, and the most socially and financially vulnerable population, have access to free maternal health services. This would improve maternal health indicators and enable progress to achieve global targets such as eight ANC contacts. With Burundi still registering more than 700 maternal deaths per 100,000 livebirths [55], an increase in the number of births attended by skilled health personnel will contribute to the Sustainable Development Goal targets of a reduction of maternal mortality ratio to less than 70 per 100,000 live births and that of neonatal mortality ratio to less than 12 per 1000 live births.

## Data Availability

Datasets and materials including STATA command file (dofile) can be obtained by sending a reasonable request to the first and corresponding author. Further, DHS datasets are publicly available on DHS program website and can be obtained upon request.

## Abbreviations

ANC: Antenatal care
DHS: Demographic and health survey
FANC: Focused antenatal care
HIV: Human immunodeficiency virus
LMICs: Low- and middle-income countries
OR: Odds ratio
WHO: World Health Organization
